# *In vivo* detection of small tumour lesions by multi-pinhole SPECT applying a ^99m^Tc-labelled nanobody targeting the Epidermal Growth Factor Receptor

**DOI:** 10.1038/srep21834

**Published:** 2016-02-25

**Authors:** Thomas Krüwel, Damien Nevoltris, Julia Bode, Christian Dullin, Daniel Baty, Patrick Chames, Frauke Alves

**Affiliations:** 1 Department of Diagnostic and Interventional Radiology, University Medical Center Goettingen, Robert-Koch-Str. 40, 37075 Goettingen, Germany; 2 Antibody therapeutics and Immunotargeting, CRCM, Inserm U1068, Institut PaoliCalmettes, Aix-Marseille Université UM 105, CNRS UMR7258, F-13009, Marseille, France; 3 Molecular Mechanisms of Tumour Cell Invasion (V077), German Cancer Research Center, Im Neuenheimer Feld 581, 69120 Heidelberg, Germany; 4 Department of Haematology and Medical Oncology, University Medical Center Goettingen, Robert-Koch-Str. 40, 37075 Goettingen, Germany; 5 Molecular Biology of Neuronal Signals, Max-Planck-Institute for Experimental Medicine, Hermann-Rein-Str. 3, 37075 Goettingen, Germany

## Abstract

The detection of tumours in an early phase of tumour development in combination with the knowledge of expression of tumour markers such as epidermal growth factor receptor (EGFR) is an important prerequisite for clinical decisions. In this study we applied the anti-EGFR nanobody ^99m^Tc-D10 for visualizing small tumour lesions with volumes below 100 mm^3^ by targeting EGFR in orthotopic human mammary MDA-MB-468 and MDA-MB-231 and subcutaneous human epidermoid A431 carcinoma mouse models. Use of nanobody ^99m^Tc-D10 of a size as small as 15.5 kDa enables detection of tumours by single photon emission computed tomography (SPECT) imaging already 45 min post intravenous administration with high tumour uptake (>3% ID/g) in small MDA-MB-468 and A431 tumours, with tumour volumes of 52.5 mm^3^ ± 21.2 and 26.6 mm^3^ ± 16.7, respectively. Fast blood clearance with a serum half-life of 4.9 min resulted in high *in vivo* contrast and *ex vivo* tumour to blood and tissue ratios. In contrast, no accumulation of ^99m^Tc-D10 in MDA-MB-231 tumours characterized by a very low expression of EGFR was observed. Here we present specific and high contrast *in vivo* visualization of small human tumours overexpressing EGFR by preclinical multi-pinhole SPECT shortly after administration of anti-EGFR nanobody ^99m^Tc-D10.

The detection of tumours in an early phase of tumour development is an important achievement to improve the overall prognosis of the patient. Besides accurate information of tumour load and spread, the retrieval of the expression of biomarkers on the tumour cell surface at the earliest time point is a prerequisite for a successful targeted therapeutic approach. In order to acquire information on expression of tumour associated proteins *in vivo*, functional imaging with specific probes targeting biomarkers such as human epidermal growth factor receptor 1 (EGFR) or 2 (HER2) is a promising approach^1^,^2^,^3^,^4^,^5^,^6^. When labelled with a radionuclide, these tracers can be detected non-invasively by positron emission tomography (PET) or single photon emission computed tomography (SPECT) with high sensitivity. The use of multi-pinhole collimators causes a magnification of the image on the detector and results in a higher resolution that is needed for preclinical imaging of small rodents^7^,^8^.

A requirement for probes to be used for tumour imaging is their fast and specific accumulation in the tumour and as little as possible uptake in healthy tissue thus generating a high contrast within the tumour shortly after probe administration. In order to achieve a fast removal from the blood pool the ideal imaging probe should be as small as possible^9^. Unlike conventional antibodies, nanobodies, also called single domain antibodies, derived from camelid heavy chain antibodies meet all these requirements with a molecular weight of only 15 kDa and dimensions of 2.5 × 4 nm^10^. Due to their small size nanobodies are removed quickly from the blood by renal clearance with half-lives in serum of less than 10 min^3^,^4^. Small-sized proteins are also known to extravasate more easily and show a better tissue penetration compared to larger molecules like full antibodies with a molecular weight of 150 kDa^10^. Due to the lack of the Fc part of an intact immunoglobulin G (IgG), nanobodies are not suspected to interfere with the immune system^11^,^12^. Furthermore, nanobodies are produced in *E.coli*, that is considered to be an economic, fast and straightforward production method with high yields. They can easily be modified with various tags e.g. hexahistidine (6 × His), myc or a free cysteine that allows a site-specific labelling for biomedical imaging.

In this study, we applied and radiolabelled the recently developed anti-EGFR nanobody D10 with ^99m^Tc-tricarbonyl species [^99m^Tc(CO)_3_]^+^ to target the EGFR that is frequently overexpressed in a variety of tumours^13^. The anti-EGFR nanobody D10 has an affinity of 7 nM towards human EGFR and does not compete with the binding site of Cetuximab, the approved anti-EGFR antibody for the treatment of different tumour entities such as colorectal or head and neck cancer (SCCHN).

Here we present non-invasive, specific and high contrast visualization of very small EGFR overexpressing human tumours with sizes ranging from 10 to 100 mm^3^ in nude mice using [^99m^Tc(CO)_3_]^+^ labelled anti-EGFR nanobody ^99m^Tc-D10 by multi-pinhole SPECT 45 min after intravenous administration.

## Results

### Radiolabeling and *in vitro* validation of the nanobodies

The anti-EGFR D10 and irrelevant control nanobody F5 were produced in *E.coli* cultures and purified via the 6 × His tags by metal affinity chromatography. For radiolabelling, the 6 × His tags of the nanobodies were site-specifically labelled with [^99m^Tc(CO)_3_]^+^ with a specific activity of 183 MBq/nmol ± 35 and 182 MBq/nmol ± 51 and a radiochemical yield of 48.8% ± 7.0 and 58.2% ± 6.7 for the anti-EGFR nanobody ^99m^Tc-D10 (N = 12) and for the control nanobody ^99m^Tc-F5 (N = 10), respectively. After purification, radiochemical purities, i.e. the amount of protein-bound activity, with values of 97.7% ± 1.2 (^99m^Tc-D10) and 98.2% ± 1.1 (^99m^Tc-F5) were determined by instant thin layer chromatography (ITLC). In comparison to the nanobodies, the anti-EGFR immunoglobulin G_1_ (IgG_1_) Cetuximab was modified with HyNic prior radiolabelling due to a missing 6 × His tag. Labelling of Cetuximab with ^99m^Tc-pertechnetate and tricine as coligand resulted in a specific activity of 1700 MBq/nmol ± 105 with a yield of 44.1 ± 10.1% and a purity of 97.3% (Table 1). The stability of the radiolabelled antibodies was tested by ITLC after incubation in mouse serum. After 4 h more than 90% of the activity remained bound to the nanobodies and 95% of the activity remained bound to IgG_1_ Cetuximab.

**Figure 1:**
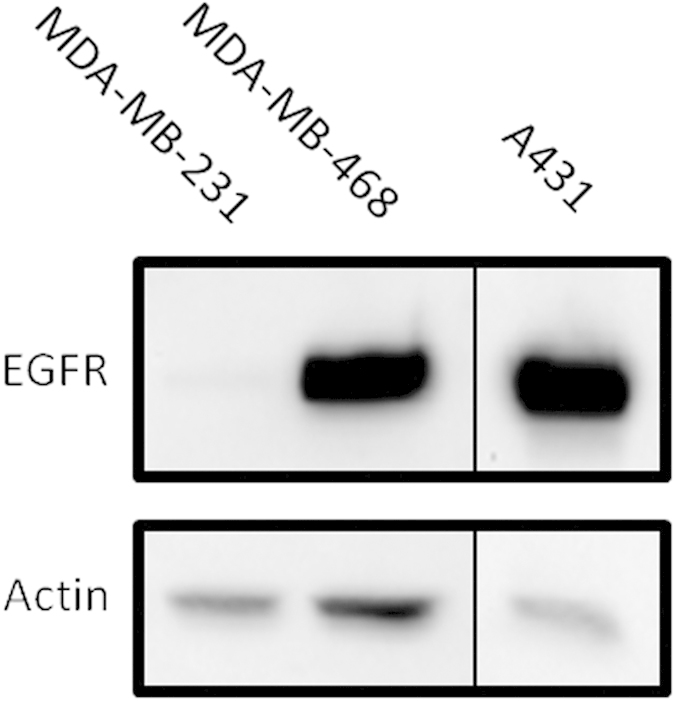
EGFR expression on human tumour cell lines. Western blot analysis of EGFR expression levels of cell lysates of human mammary carcinoma cell lines MDA-MB-231 and MDA-MB-468 as well as of the human epidermoid carcinoma cell line A431 are shown. A high EGFR expression is found in MDA-MB-468 and A431 cells in comparison to MDA-MB-231 cells, which only express low amounts of EGFR. Actin was used as loading control.

The protein expression levels of EGFR on cells of the human mammary carcinoma cell lines MDA-MB-231 and MDA-468 as well as of the human epidermoid carcinoma cell line A431 were investigated by Western blotting. Figure 1 depicts a low EGFR expression level for MDA-MB-231 and a high expression level for MDA-MB-468 and A431 cells.

**Figure 2:**
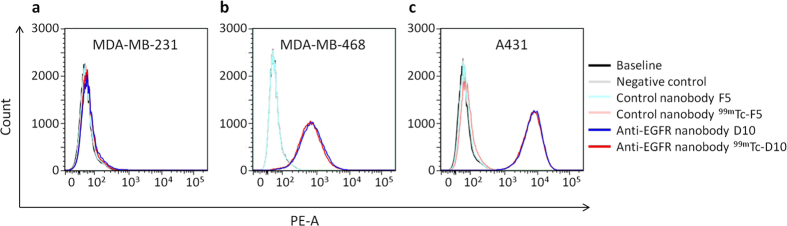
Specific binding of anti-EGFR nanobody D10 to tumour cells. Binding capacities of anti-EGFR nanobody D10 (blue) and radiolabelled ^99m^Tc-D10 (red) on (**a**) mammary carcinoma cells MDA-MB-231 and (**b**) MDA-MB-468 as well as (**c**) epidermoid carcinoma cells A431 were investigated by flow cytometry and compared to the irrelevant control nanobody F5 (light blue) and to the radiolabelled ^99m^Tc-F5 (light red). Results demonstrate no alteration of the binding properties to EGFR caused by the radiolabelling procedure. All nanobodies were revealed by consecutive incubation with an anti-myc antibody and goat-anti-mouse antibody coupled to R-Phycoerythrin (PE). Mean Fluorescence Intensity (MFI) of control nanobody F5 matched the MFI values of background and negative control (i.e. incubation with anti-myc antibody and goat-anti-mouse-PE only), ruling out any unspecific binding to the tumour cells. 5 × 10^5^ cells per sample were recorded and MFIs were displayed as histograms.

Binding properties of the unlabelled nanobodies D10 and F5 were compared to the ones of radiolabelled nanobodies ^99m^Tc-D10 and ^99m^Tc-F5 on endogenously expressed EGFR on A431, MDA-MB-231 and MDA-MB-468 tumour cells using flow cytometry after decay of the radioactivity. In all three cell lines no difference in the mean fluorescence intensity (MFI) between the radiolabelled ^99m^Tc-D10 and ^99m^Tc-F5 (red line) and unlabelled (blue line) nanobodies D10 and F5 could be detected indicating that ^99m^Tc-D10 still bound the same amount of EGFR on cells as D10 (Fig. 2). These results demonstrate no alteration of the binding properties to EGFR caused by the radiolabelling procedure. As expected, irrelevant nanobodies F5 and ^99m^Tc-F5 showed no binding on all cell lines (light blue and red lines) ruling out any unspecific binding of nanobodies to the investigated tumour cells (Fig. 2). The MFI of the anti-EGFR nanobody D10 correlated well with the receptor density on the cell surface of A431 and MDA-MB-468 cells, both characterized by a high EGFR expression (Fig. 2b,c) and MDA-MB-231 by a low EGFR expression (Fig. 2a) as shown in Western blot analysis (Fig. 1). The EGFR expression level on MDA-MB-231 cells represents the detection limit for the anti-EGFR nanobody D10 since no increase of the MFI compared to baseline could be detected under these conditions.

### Nanobodies targeting EGFR allow a specific high contrast tumour detection *in vivo* 45 min after administration

**Figure 3:**
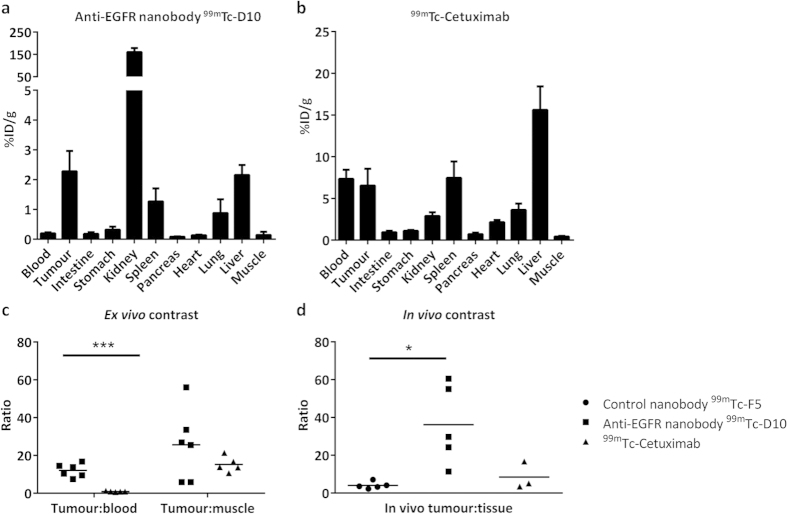
Uptake of anti-EGFR nanobody ^99m^Tc-D10 in A431 tumours in comparison to ^99m^Tc-Cetuximab. A431 tumour bearing mice received either 17 pmol (2.6–5.1 MBq) radiolabelled anti-EGFR nanobody ^99m^Tc-D10 (N = 6) or 9 pmol (9.4–15.6 MBq) ^99m^Tc-Cetuximab (N = 5) intravenously. *Ex vivo* biodistribution (**a**) 100 min post injection of anti-EGFR nanobody ^99m^Tc-D10 and (**b**) 25 h post injection of ^99m^Tc-Cetuximab are shown. (**c**) *Ex vivo* tumour to blood ratios and tumour to tissue (muscle) ratios after nanobody ^99m^Tc-D10 application (100 min post injection) compared to ^99m^Tc-Cetuximab (25 h post injection) are presented. (**d**) *In vivo* SPECT scans were performed 45 min post nanobody ^99m^Tc-D10 and irrelevant nanobody ^99m^Tc-F5 injection and 24 h post ^99m^Tc-Cetuximab injection. The area of the tumour was segmented and compared to an equally sized contra lateral region as a measure for tissue uptake. Ratios were calculated as ratio of tumour uptake and tissue uptake. *P < 0.05, unpaired two-tailed t-Test, for *in vivo* tumour to tissue ratio: nanobody ^99m^Tc-D10 vs. irrelevant nanobody ^99m^Tc-F5 and nanobody ^99m^Tc-D10 vs. ^99m^Tc-Cetuximab. ***P < 0.001, unpaired two-tailed t-Test, for *ex vivo* tumour to blood ratio: nanobody ^99m^Tc-D10 vs. ^99m^Tc-Cetuximab.

**Figure 4:**
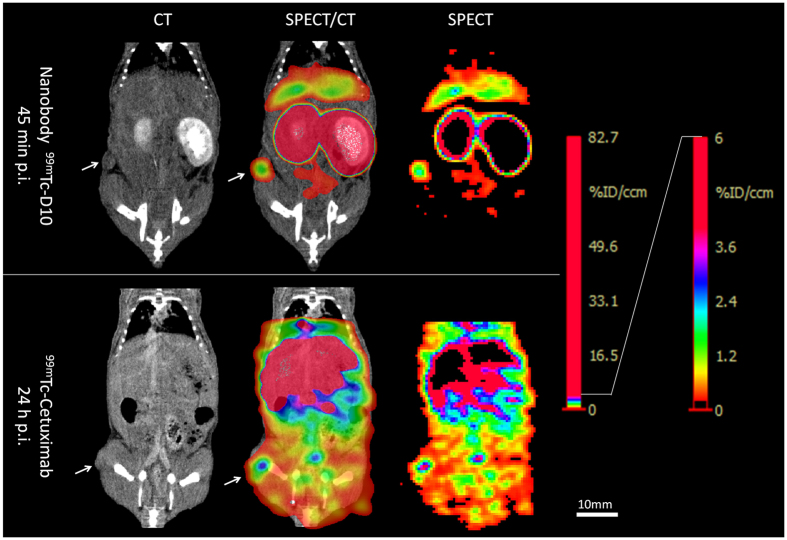
*In vivo* tumour visualization of small human A431 tumour xenografts with anti-EGFR nanobody ^99m^Tc-D10 in comparison to ^99m^Tc-Cetuximab by SPECT and CT. A431 tumour bearing mice received 17 pmol (3.3 MBq; tumour volume 22 mm^3^) radiolabelled anti-EGFR nanobody ^99m^Tc-D10 or 9 pmol (10.5 MBq; tumour volume 16 mm^3^) ^99m^Tc-Cetuximab intravenously. SPECT imaging was performed 45 min post ^99m^Tc-D10 and 24 h post ^99m^Tc-Cetuximab administration. Contrast-enhanced CT and SPECT scans were performed on different modalities and images were aligned by hand according to ^99m^Tc-pertechnetate landmarks (<30 kBq). Tumours are indicated by white arrows. Note, that a high tumour accumulation with a low background was achieved with anti-EGFR nanobody ^99m^Tc-D10.

Mice bearing human A431 epidermoid subcutaneous tumour xenografts of small size with a mean tumour volume of 26.6 mm^3^ (N = 6; range 7-54 mm^3^; Table 2) received the anti-EGFR nanobody ^99m^Tc-D10 that accumulated in the tumour and generated a high tumour to tissue contrast determined by *in vivo* and *ex vivo* biodistribution analysis. Due to a fast blood clearance with a serum half-life of 4.9 min *in vivo* SPECT imaging was performed already 45 min post probe injection. SPECT imaging revealed a good contrast with tumour to tissue (area of the contra lateral side) ratios of 36.2 ± 20.9 in 5 out of 6 animals (Figs 3d and 4). Besides tumour derived signals, the kidneys and the liver were clearly visible by SPECT imaging (Fig. 4). The specific tumour uptake was validated in previously performed SPECT scans in the same mice by applying the control nanobody F5 that showed no tumour uptake in all mice (N = 5, Fig. 3d). Following *in vivo* SPECT imaging, the uptake of anti-EGFR nanobody ^99m^Tc-D10 in A431 tumours was confirmed by *ex vivo* biodistribution analyses in all six animals with an uptake of 2.27% ID/g ± 0.68 (Fig. 3a) resulting in a tumour to blood ratio of 12.1 ± 3.5 and a tumour to tissue (muscle) ratio of 25.6 ± 18.8 (Fig. 3c, Table 2). In kidneys and liver uptakes of 160.7% ID/g ± 17.9 and 2.1% ID/g ± 0.3 were determined, respectively. These findings result in tumour to kidney and tumour to liver ratios of 0.014 ± 0.004 and 1.1 ± 0.4, respectively, demonstrating that the nanobody ^99m^Tc-D10 was excreted via the kidney.

A resolution of more than 2 mm was determined for the multi-pinhole collimators used here by SPECT scans with a Jaszczak phantom corresponding to a volume of at least 8 mm^3^ (data not shown). Therefore, tumours with volumes below 8 mm^3^ cannot be detected by the here used SPECT system. This explains the absent signals within the tumour of approximately 7 mm^3^ in the SPECT scans of one out of six mice. Although this very small tumour was not visualized by *in vivo* SPECT due to the limited resolution, a distinct uptake of up to 2.18% ID/g of ^99m^Tc-D10 was proven by *ex vivo* biodistribution.

The efficacy of *in vivo* tumour detection with anti-EGFR nanobody ^99m^Tc-D10 was compared to the IgG_1_^99m^Tc-Cetuximab that was used to visualize A431 tumour lesions with comparable mean tumour volumes of 40 mm^3^ (N = 5; range 7–90 mm^3^) 45 min post injection. *In vivo* SPECT scans showed no uptake of ^99m^Tc-Cetuximab in the small tumour lesions at the early time point. It was not possible to define the tumour at the early time point due to the high background in any of the scans performed 45 min post injection. High uptakes were found in all organs and large blood vessels like the inferior vena cava and the external iliac veins (Supplementary Fig. S1). However, in a second *in vivo* SPECT scan 24 h post injection (Fig. 4), a tumour uptake in 3 out of 5 animals could be detected with a tumour to tissue ratio of 8.4 ± 7.2 (Fig. 3d, Table 2), much less compared to results obtained with the nanobody ^99m^Tc-D10. Of note are the high and low background signals obtained by application of ^99m^Tc-Cetxuimab and ^99m^Tc-D10, respectively (Fig. 4).

*Ex vivo* biodistribution analysis performed 100 min post injection revealed a tumour uptake of 6.5% ID/g ± 2.0 (Fig. 3b) for ^99m^Tc-Cetuximab resulting in a tumour to blood ratio of only 0.9 ± 0.3 and a tumour to tissue ratio of 15.2 ± 4.1 (Fig. 3c, Table 2). This represents one tenth of the tumour to blood ratio and half of the tumour to tissue ratio obtained with the anti-EGFR nanobody ^99m^Tc-D10.

### Detection of tumours is dependent of EGFR expression

**Figure 5:**
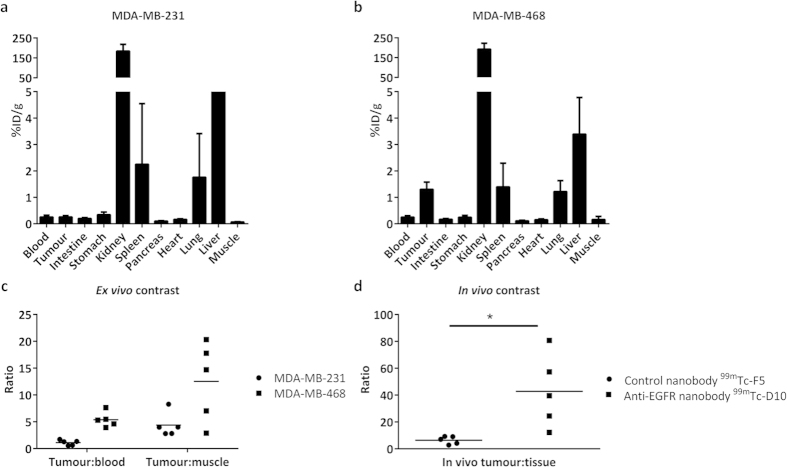
Tumour uptake of anti-EGFR nanobody ^99m^Tc-D10 is dependent on EGFR expression. MDA-MB-231 and MDA-MB-468 tumour bearing mice received 17 pmol (2.3 MBq) anti-EGFR nanobody ^99m^Tc-D10 and 17 pmol of irrelevant nanobody ^99m^Tc-F5 (2.4 MBq) intravenously. (**a**) *Ex vivo* biodistribution 100 min post injection of anti-EGFR nanobody ^99m^Tc-D10 are shown in MDA-MB-231 (N = 5) and (**b**) MDA-MB-468 (N = 5) tumour bearing mice. (**c**) *Ex vivo* tumour to blood ratios and tumour to tissue (muscle) ratios of nanobody ^99m^Tc-D10 are presented. (**d**) *In vivo* SPECT scans were performed in MDA-MB-468 tumour bearing mice 45 min post injection of either nanobody ^99m^Tc-D10 or control nanobody ^99m^Tc-F5. The area of the tumour was segmented and compared to an equally sized contra lateral region as measure for tissue uptake. *P < 0.05, unpaired two-tailed t-Test, for *in vivo* tumour to tissue ratio nanobody ^99m^Tc-D10 vs. control nanobody ^99m^Tc-F5.

In order to preclinically assess the efficacy of anti-EGFR nanobody ^99m^Tc-D10 to detect small tumours in different tumour entities, MDA-MB-468 and MDA-MB-231 human mammary carcinoma cells were implanted orthotopically in the fat pad of the right abdominal mammary gland of nude mice. Mice bearing high EGFR expressing MDA-MB-468 tumours with a mean volume of 56.5 mm^3^ ± 21.2 (N = 5; range 35.1–89.4 mm^3^) were used for *in vivo* tumour detection with anti-EGFR nanobody ^99m^Tc-D10. All MDA-MB-468 tumours were detected reliably by *in vivo* SPECT scans 45 min after administration of 17 pmol anti-EGFR nanobody ^99m^Tc-D10 as shown by signals present in the tumour and very low background signals resulting in a tumour to tissue (area of the contra lateral side) ratio of 42.8 ± 27.0 (Fig. 5d, Table 2). *Ex vivo* analyses of biodistribution of MDA-MB-468 tumour bearing mice revealed a tumour uptake of 1.3% ID/g ± 0.27 (Fig. 5b) with a tumour to blood ratio of 5.4 ± 1.4 and a tumour to tissue ratio of 12.5 ± 7.3 (Fig. 5c, Table 2). The kidneys and the liver showed an uptake of 192.5% ID/g ± 30.9 and 3.4% ID/g ± 1.4 of ^99m^Tc-D10, respectively (Fig. 5b, Table 2).

**Figure 6:**
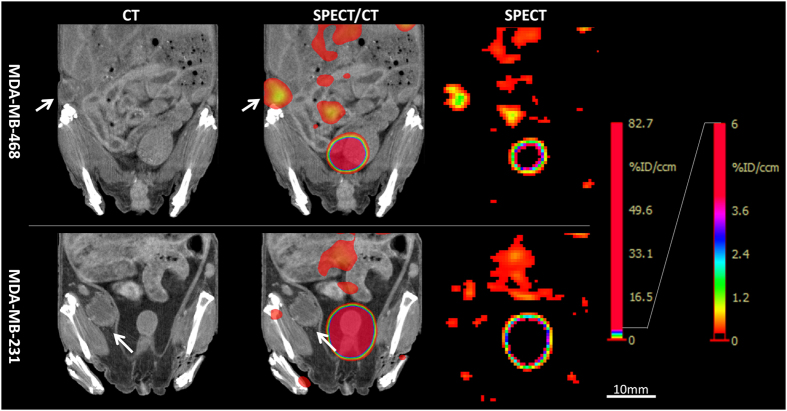
*In vivo* tumour visualization of human orthotopic mammary MDA-MB-468 and MDA-MB-231 tumours with anti-EGFR nanobody ^99m^Tc-D10 by SPECT and CT is dependent on EGFR expression. Representative images of mice bearing either an orthotopically implanted MDA-MB-468 tumour (volume 64 mm^3^) or MDA-MB-231 tumour (volume 129 mm^3^) that received 17 pmol (2.4–3.1 MBq) anti-EGFR nanobody ^99m^Tc-D10 by intravenous administration, are shown. SPECT imaging was performed 45 min post injection. Contrast-enhanced CT (left panel) and SPECT scans (right panel) were performed on different modalities and images were aligned by hand according to ^99m^Tc-pertechnetate landmarks (middle panel). Tumours are indicated by white arrows. A high tumour accumulation with a low background was achieved with anti-EGFR nanobody ^99m^Tc-D10 in the MDA-MB-468 tumour model. Accumulation of nanobody ^99m^Tc-D10 was not detectable in the MDA-MB-231 tumour model with a very low EGFR expression.

MDA-MB-231 tumours with a very low expression of EGFR showed no detectable uptake of the anti-EGFR nanobody ^99m^Tc-D10 (Fig. 6) *in vivo*. Neither *in vivo* SPECT (Fig. 6) nor *ex vivo* biodistribution analyses (Fig. 5a, Table 2) demonstrate an uptake of ^99m^Tc-D10 in tumours with a mean volume of 124 mm^3^ ± 102 (N = 5; range 21.1–247.5 mm^3^). The lack of ^99m^Tc-D10 to accumulate within the tumour indicated no unspecific binding or tumour uptake.

**Figure 7:**
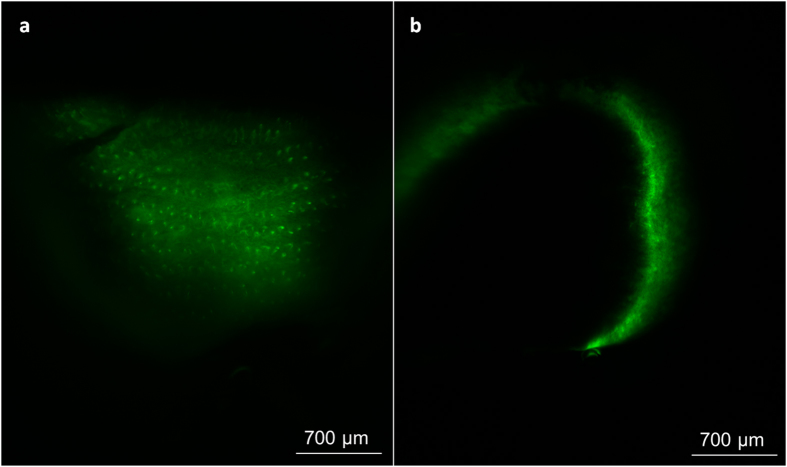
Homogenous tumour penetration of the anti-EGFR nanobody D10 in an A431 tumour. Anti-EGFR nanobody AF488-D10 (14 pmol) was injected in the tail vein of a nude mouse bearing a A431 tumour. (**a**) depicts an overview at 4x magnification of a slice in the middle of the tumour that shows the nanobody AF488-D10 binding on single cells and (**b**) shows a slice of half of the spleen in an upright position in 175 μm depth at 4x magnification. Due to the dehydration and clearing procedure the volume of the spleen was decreased but subcellular structures remained unmodified. Only background fluorescence and no distinct structures can be observed within the spleen.

To assess the distribution of anti-EGFR nanobody D10 through the tumour tissue, a small high EGFR expressing A431 tumour with a volume of 6.6 mm^3^ was investigated by single plane illumination microscopy (SPIM) 45 min after intravenous administration of anti-EGFR nanobody AF488-D10. This allows the analysis of whole tumour lesions and provides the possibility to three-dimensionally acquire the tumour tissue penetration of nanobody D10 labelled to AlexaFluor 488 (AF488-D10) on a cellular resolution^14^. In order to validate the results obtained with nanobody AF488-D10 in tumour tissue, the spleen of the same mouse was scanned to determine background fluorescence. At a depth of 175 μm of the surface, the fluorescence signal was distributed through the whole tumour. Many circular structures that might correspond to the shape of single cells were visible in the middle of the tumour suggesting a homogenous distribution within the tumour tissue and binding of nanobody AF488-D10 to single tumour cells (Fig. 7a).

A movie of the whole z-stack is provided in the Supplementary Video S2 showing several layers of tumour cells with bound AF488-D10. Figure 7b depicts an image obtained by SPIM of a part of the spleen that serves as negative control. Only unspecific background fluorescence and no visible cell structures can be observed. Therefore, the signals in the tumour suggest a specific binding of nanobody AF488-D10 to single cells in the tumour.

## Discussion

In this study we demonstrate that the use of anti-EGFR nanobody ^99m^Tc-D10 is especially suitable to detect small tumour lesions expressing EGFR with high specificity and high contrast 45 min after administration. These features are mandatory for tracers to enable the detection of tumours with small sizes at earliest time points to improve the overall prognosis for the patient. *In vivo* SPECT scans were performed already after 45 min in tumour lesions expressing high levels of EGFR with an excellent contrast with low background signals resulting in a tumour to tissue ratio ranging from 11 to up to 80. This early detection was feasible due to the fast blood clearance with a half life in blood of only 4.9 min and a very low plateau of 0.26% ID/g ± 0.24 of the remaining activity in the blood pool.

Here, we show that the anti-EGFR nanobody ^99m^Tc-D10 is suitable to visualize small tumour lesions with weights below 100 mg corresponding to tumour volumes below 100 mm^3^. It is known that most tumours originate as small avascular structures and have to establish their own tumour derived blood vessels to grow beyond a few millimetres^15^,^16^. Thus, we assume that the small-sized tumours analysed here do not have high amounts of vasculature with fenestrated blood vessels that allow a quick extravasation of the nanobody and thereby a higher accumulation of probes within the tumour as described for larger tumours^15^,^16^,^17^,^18^,^19^,^20^,^21^. The successful tumour visualization with high uptakes of the anti-EGFR nanobody ^99m^Tc-D10 can be explained by the small size of the nanobody enabling a good tissue penetration. Recent studies have shown that smaller antibodies are characterized by a better penetration through the tissue^10^,^22^. It has been shown by others that in small tumour lesions with a tightly sealed vasculature the nanobodies will most likely extravasate along the negative pressure gradient between blood vessels and interstitial space which results in a drainage towards the interstitial space and further to the lymphatic ducts suggesting that tumour targeting occurs by diffusion^10^. The fact that the unbound nanobody ^99m^Tc-D10 is quickly removed from the tissue as well as from the blood resulted in this study in a high tumour contrast with high *in vivo* tumour to tissue and *ex vivo* tumour to muscle ratios.

Nanobodies are subject of investigation for several years and many studies were published targeting EGFR or HER2 overexpressing tumours, however mainly tumours with diameters of approximately 0.5–1 cm^3^,^23^ or volumes of above 100 mm^3^ with tumour weights far beyond 100 mg^3^,^4^,^23^,^24^. These nanobodies have comparable affinities in the nanomolar range for their target receptors and were cleared within minutes to hours from the blood pool. The overall values for A431 tumour uptake were 2–3 fold higher *in vivo* as well as *ex vivo* for the published anti-EGFR nanobodies^3^,^4^,^23^. In our study with ^99m^Tc-D10, we found lower background activities in the blood (*ex vivo*) as well as in the tissue (*in vivo*) compared to the reported anti-EGFR nanobodies^3^,^4^,^23^. A reason for the higher tumour uptake of previous reports using nanobodies might be the larger diameter (0.5–1 cm) or volume (100–600 mm^3^) of tumours used in these studies, leading to better vascularisation of the tumour with more fenestrated blood vessels^3^,^4^. A431 tumours with a volume of at least 100 mm^3^ were shown to contain many small and mostly immature blood vessel that were homogenously distributed throughout the tumour^25^. Contrary to our study, Gainkam *et al.* reported an inverse relationship between tumour uptake and tumour weight with EGFR targeting in A431 tumours with different anti-EGFR nanobodies^3^. The tendency of decreasing tumour uptake with increasing tumour weight was reported for tumours with weights ranging from 120 mg to above 800 mg^3^. This effect can be explained on one hand by intratumoural heterogeneity and on the other hand by the diameter of the blood vessels that becomes narrower from the periphery to the centre and might reduce the amount of nanobody reaching the centre^25^. A third explanation might be the formation of necrotic tissue in the centre of tumours of big sizes that do not bind nanobodies.

The full IgG_1_^99m^Tc-Cetuximab led to a significantly lower tumour to blood ratio compared to nanobody ^99m^Tc-D10, despite a more than 10 fold higher affinity towards EGFR (0.5 nM)^26^. Similarly, the *ex vivo* tumour to blood ratio was significantly impaired (P < 0.001) for ^99m^Tc-Cetuximab after 25 h compared to ^99m^Tc-D10 after 100 min. Targeting of EGFR with ^99m^Tc-Cetuximab in our setting resulted in a relatively low overall A431 tumour uptake of 6.5% ID/g compared to tumour uptakes of up to 20% ID/g observed by others^20^,^21^. With ^99m^Tc-Cetuximab we observed a long serum half-life (approx. 3 h in this murine model) which can be explained by the relatively large size of the IgG_1_, 152 kDa, and its removal from the body via hepatic excretion, together with the recycling process in the cells of the liver afforded by the binding of its Fc portion to the FcRn receptor^27^,^28^. Since a high kidney uptake of approx. 150% ID/g of ^99m^Tc-D10 was observed in our study the anti-EGFR nanobody ^99m^Tc-D10 is predominantly removed by renal excretion. High uptakes in the kidneys have previously been reported for many other nanobodies^3^,^4^,^10^,^23^,^24^,^29^,^30^,^31^ and excretion via the kidney is described for small proteins with a molecular weight of 15 kDa^3^,^4^,^6^,^32^, a value under the 60 kDa threshold of glomerular filtration in the kidneys^33^,^34^,^35^.

The renal uptake is mediated by the Low Density Lipoprotein receptor-related protein 2 (LRP2 or megalin) in the proximal tubular cells^23^. Megalin is known to recover proteins from the urine by interaction on cationic domains via endocytosis or transcytosis to prevent proteinuria^36^,^37^. This unspecific accumulation might be problematic for imaging tumour lesions in close proximity to the kidneys and for the use of the nanobody as carrier for therapeutic applications such as the conjugation of beta emitters to nanobodies for radioimmunotherapy. A possibility to reduce this renal uptake is the coinjection of cationic or polycationic amino acids or succinylated gelatine (gelofusine)^23^,^33^,^34^,^35^. Interestingly, Chatalic *et al.* recently reported that the removal of the C-terminal His tag from an anti-PSMA nanobody also markedly reduced its renal uptake and could be combined with gelofusine and lysine to decrease the renal uptake below 4% ID/g at 3 h post injection^38^.

For a successful tumour visualization, high contrast is needed to define the tumour from the surrounding tissue. Here, by the use of *in vivo* SPECT with ^99m^Tc-D10 tumours could be clearly visualized with a very low background resulting in high tumour to tissue ratios.

The variations of tumour to tissue ratios are large compared to the data of *ex vivo* biodistribution analysis. The uptake of ^99m^Tc-D10 in the muscle of A431 tumour bearing mice was very low with 0.14% ID/g ± 0.1, however, with a distinctive variance. This variance is even more pronounced after calculation of the ratio. For ^99m^Tc-Cetuximab, this effect was less marked, since the muscle uptake was 3-fold higher, and varied to a lesser extent with values of 0.43% ID/g ± 0.08 compared to ^99m^Tc-D10. The lower variation in this experimental set up can be explained by the late time point of the *in vivo* SPECT scan at 24 h post ^99m^Tc-Cetuximab injection. The variation of the ratios did not interfere with interpretation and analysis of images since sufficient contrast between tumour and muscle was provided.

Our results also illustrate specific tumour detection of nanobody ^99m^Tc-D10 to EGFR expressing tumour cells since no tumour uptake *ex vivo* and consequently no tumour uptake *in vivo* could be observed in the low EGFR expressing MDA-MB-231 tumour model.

Our study shows that ^99m^Tc-D10 represents a versatile tool for the specific detection of small EGFR overexpressing tumour lesions and the assessment of EGFR expression in tumours by *in vivo* SPECT. Of crucial importance, we show the possibility to acquire high contrast images with a high tumour to background ratio shortly after probe administration, i.e. allowing imaging at the same day. Therefore, anti-EGFR nanobody ^99m^Tc-D10 might be suitable in a clinical setting as non-invasive diagnostic tracer to not only detect small tumours but also to obtain information on the expression level of EGFR at the time of diagnosis and during disease progression.

## Experimental Section

### Expression of nanobodies in *E. coli*

Selection and screening of the anti-EGFR nanobody D10 was published recently^13^. The irrelevant nanobody F5 was selected according to the protocol of D10, but does not bind to EGFR. Freshly transformed *E. coli* BL21-DE3 were grown overnight in 400 ml 2 YT medium. Nanobodies were harvested by lysis of the bacteria with BugBugster extraction reagent (MerckMillipore, Schwalbach, Germany) supplemented with Lysozyme and Benzonase, and purified by IMAC (Talon SuperFlow, GE Healthcare, Freiburg, Germany).

### Cell culture

Human mammary carcinoma cell lines MDA-MB-231 and MDA-MB-468 and human epidermoid carcinoma cell line A431 (DSMZ, Braunschweig, Germany) were cultured in high glucose (4.5 g/l) DMEM with Glutamax (LifeTechnologies, Darmstadt, Germany) supplemented with 10% fetal calf serum (PAA, Cöble, Germany). All cells were grown in monolayer at 37 °C, 5% CO_2_ in a humidified atmosphere and detached with 0.05% trypsin/EDTA (Biochrom, Berlin, Germany) at a subconfluent stage.

### Western blot

Tumour cells were seeded on 6 cm petri dishes and allowed to grow to a subconfluent stage, washed with cold phosphate buffered saline (PBS) and lysed with radioimmunoprecipitation assay (RIPA) buffer (Sigma Aldrich, Schelldorf, Germany). For Western blot analysis, 25 μg total lysate were separated by sodiumdodecylsulfate polyacrylamide gel electrophoresis (3–8% gel, Novex, LifeTechnologies, Darmstadt, Germany) and blotted on nitrocellulose membrane. Primary antibodies targeting EGFR (#2232) and Actin (#MAB1501) were purchased from Cell Signaling (New England Biolabs, Frankfurt a.M., Germany) and MerckMillipore (Schwalbach, Germany), respectively. HRP-labelled secondary anti-mouse (#NA931VS) and anti-rabbit (#NA934VS) antibodies were purchased from Amersham (GE Healthcare, Freiburg, Germany). Proteins were detected by brief incubation with the ECL detection kit (Amersham) and by acquisition on a ChemiDoc XRS system (BioRad, München, Germany).

### Radiolabeling of nanobodies and IgG

The nanobodies were labelled with technetium-99m tricarbonyl ([^99m^Tc(CO)_3_]^+^) species via their C-terminal hexahistidine tags (6 × His). The CRS Kit (Paul Scherrer Institut, Villingen, Switzerland) was used to produce the [^99m^Tc(CO)_3_]^+^ intermediate out of 2400–4000 MBq of ^99m^Tc pertechnetate in 1 ml saline. After boiling for 20 min at 100 °C, 500 μl of [^99m^Tc(CO)_3_]^+^ were neutralized with 1 M HCl to pH 7.5 and incubated with 50 μg nanobody (1 mg/ml) for 90 min at 56 °C. The radiolabeld nanobodies were purified from unbound activity on Amicon Ultra centrifugal filters (Merck Millipore, Schwalbach, Germany).

For radiolablelling of the full IgG_1_ Cetuximab (Merck, Darmstadt, Germany), the free ε-amino groups were modified with 6-hydrazinonicotinamide (HyNic). 10 nmol antibody were incubated with an 30 molar excess of succinimidyl 6-hydraziniumnicotinate hydrochlorate (SHNH) (SoluLink, Biotrend, Cologne, Germany) for 2 h at ambient temperature in a 100 mM phosphate buffer (pH 7.4). 20 μl of the antibody-HyNic conjugate (2 mg/ml) in 100 mM phosphate buffer (pH 6.0) were incubated with 500 μl 99mTc pertechnetate (1000–2000 MBq), SnCl_2_ (5 μl; 1 mg/ml; Sigma Aldrich, Schnelldorf, Germany) and tricine (50 μl; 100 mg/ml; Sigma) as coligand for 20 min at ambient temperature. The radiolabelled Cetuximab was purified from unbound activity on Amicon Ultra centrifugal filters (Merck Millipore, Schwalbach, Germany). The radiochemical yield and purity of the radiolabelling procedures were determined by instant thin layer chromatography on iTLC-SG strips (Agilent, Lake Forest, CA) and 1% HCl in methanol as mobile phase.

### Serum stability studies

Nanobodies ^99m^Tc-D10 and ^99m^Tc-F5 and IgG_1_^99m^Tc-Cetuximab were labelled with ^99m^Tc as described above. 2–3 μg of the radiolabelled proteins were mixed with mouse serum and incubated at 37 °C. Samples were taken at 0, 30, 60, 120 and 240 min and analyzed by thin layer chromatography using iTLC-SG strips and 1% HCl in methanol as mobile phase.

### Site-specific labelling of the nanobody with the fluorescent dye AlexaFluor 488

The anti-EGFR nanobody D10 was produced with a free terminal cysteine as described previously^28^. The thiol-groups of the nanobody (100 μM) in phosphate buffer (50 mM, pH 7) were reduced by a 10 fold Molar excess of DTT (10 mM) under inert atmosphere for 30 min at ambient temperature. DTT was removed by dialysis on Zeba Desalting columns (7 MWCO, Thermo Scientific, Darmstadt, Germany). A 10-fold Molar excess of AlexaFluor 488 C5-maleimide (10 mM; LifeTechnologies, Darmstadt, Germany) was added drop wise to the reduced nanobody and incubated for 2 h at ambient temperature under inert atmosphere. The reaction was stopped by the addition of an excess Glutathione. The fluorescent labelled nanobody D10 was purified from unbound dye by dialysis.

### Flow cytometry

Binding capacities of the labelled and unlabelled nanobodies were assessed by flow cytometry. Cells were detached by brief incubation with trypsin/EDTA, washed and counted. 1 × 10^6^ cells were blocked in 2% bovine serum albumin (BSA) (Sigma Aldrich, Schnelldorf, Germany) in PBS and incubated with 100 μl nanobody or antibody diluted in 2% BSA (10 μg/ml) for 30 min on ice. The nanobodies were detected by consecutive incubation with an anti-myc antibody (4 μg/ml; 2% BSA) (clone 9E10) and goat-anti-mouse-PE (4 μg/ml; 2% BSA) (both from Santa Cruz, Heidelberg, Germany) for 30 min on ice. Fluorescence intensities were measured by flow cytometry on a FACSAria cell sorter with FACSDiva software (BD Bioscience, Heidelberg, Germany). Data were analyzed using FlowJo software (v7.6.5, TreeStar Inc., Ashland, OR).

### Tumour mouse models

All animal experiments were carried out in accordance with the German animal welfare law and were approved by local authorities (Lower Saxony State Office for Consumer Protection and Food safety–LAVES). Cells of the human epidermoid carcinoma cell line A431 (1.5 × 10^6^ in 100 μl PBS) were injected subcutaneously in the right flank of 8–12 weeks old male athymic nude mice (NMRI-Foxn1) (Charles River, Sulzfeld, Germany) under control of 2% isoflurane. Cells of the human mammary carcinoma cell lines MDA-MB-231 (1 × 10^6^ in 20 μl PBS) and MDA-MB-468 (2 × 10^6^ in 20 μl PBS) were implanted orthotopically in the fat pad of right abdominal mammary gland of 8–12 weeks old female athymic nude mice (NMRI-Foxn1) (Charles River, Sulzfeld, Germany) under the control of a general Ketamine/Rompun anaesthesia as described previously^39^,^40^.

### *In vivo* imaging

All tumour volumes were determined *in vivo* by contrast-enhanced computed tomography (CT) scans under 1% isoflurane anaesthesia. 100 μl Ultravist370 (Bayer, Leverkusen, Germany) were injected intravenously and two 360° scans were carried out on a low-dose *in vivo* small animal CT (Quantum FX, Perkin Elmer, Waltham, MA) with the following parameter: 90 kV, 200 μA and a field of view of 40 (2 min total scan time; 1 min post injection) and 73 mm (17 sec total scan time; 5 min post injection). Image reconstruction was performed using a standard filtered backprojection algorithm implemented in the vendors software resulting in data sets with a voxel size of either ~80 × 80 × 80 μm^3^ or 140 × 140 × 140 μm^3^.

One to two days after CT imaging, SPECT was performed on a triple-head clinical gamma camera (XP3000, Picker) equipped with multi-pinhole collimators with 6 pinholes and a field of view of 50 × 60 mm (HiSPECT, SciVis, Göttingen, Germany) on tumour bearing mice under 1% isoflurane anaesthesia. SPECT scans of mice that received radiolabelled nanobodies were started 45 min post intravenous injection of ^99m^Tc-F5 and ^99m^Tc-D10 (14 pmol; 2.1–5.7 MBq) in the tail vein. Mice that received radiolabelled ^99m^Tc-Cetuximab (9 pmol; 9.4–15.6 MBq) were started 45 min and 24 h post intravenous injection. Images were acquired over 360° in 10 projections of 300 sec each into 256 × 256 matrices, resulting in a total scan time of 50.6 min. SPECT scans with the anti-EGFR nanobody ^99m^Tc-D10 and the control nanobody ^99m^Tc-F5 were performed in the same animals using identical scan protocols. ^99m^Tc-D10 was injected intravenously at least 72 h after administration of the control nanobody ^99m^Tc-F5.

Phantom scans were conducted with a custom made Jaszczak phantom with rod diameters ranging from 1.0 to 2.0 mm. The phantom was filled with approximately 2 ml [99mTc]NaTcO_4_ and scanned with the *in vivo* scan protocol as described above.

Image reconstruction was performed using an ordered-subset expectation maximization algorithm implemented in the HiSPECT software (SciVis, Göttingen, Germany) resulting in data sets with a voxel size of 600 × 600 × 600 μm^3^. Since CT and SPECT scans were performed on separate systems on different days, each mouse was imaged in the same animal holders, which included 6 holes each filled with ^99m^Tc-pertechnetate (<30 kBq) that were used for manual alignment.

### Image analysis

Reconstructed CT and SPECT data sets were quantified and analyzed for determination of tumour volume and tumour uptake using Scry v. 5.0 (Kuchel & Sautter GbR, Bad Teinach, Germany).

SPECT data were decay corrected and normalized. All tumour volumes were segmented from contrast enhanced CT and SPECT images by a region growing algorithm. For reference a region with the same size like the tumour containing tissue only has been segmented on the contra lateral side and was used for determination of *in vivo* tumour to tissue ratio. Tumour and tissue uptake were expressed as percent of injected dose per cubic centimeter (% ID/cm^3^).

### Biodistribution analysis

Following SPECT scans, mice were sacrificed by cervical dislocation under isoflurane anesthesia. The organs were dissected, blotted dry and weighed. Remaining activity in organs and tumours was measured in a gamma counter (Wallac Wizard 3″ 1480 automatic gamma counter; Perkin Elmer, Waltham, MA), decay corrected and expressed as percent injected dose per gram (% ID/g). The total injected dose of each mouse was determined by measuring the activity in the syringes before and after injection.

### Single Plane Illumination Microscopy (SPIM)

The anti-EGFR nanobody D10 was labelled site-specifically with a AlexaFluor 488-maleimido dye on the free cysteine. 14 pmol of the anti-EGFR nanobody AF488-D10 were injected in the tail vein of a xenografted A431 cancer xenografted nude mouse. The mouse was sacrificed 45 min post injection and the tumour and the spleen were dissected, fixed in 4% paraformaldehyde (PFA) (Sigma Aldrich, Steinheim, Germany) and sectioned coronally in cold PBS. Slices were collected in PBS in 6 well plates and removed individually for clearing. For SPIM analysis tumour tissue was optically cleared at room temperature with organic solvents using an altered 3DISCO protocol as described previously^41^. A431 tumour tissue and spleen were transferred into glass vials and the first clearing solution, 50% Tetrahydrofuran (THF; Sigma Aldrich, Steinheim, Germany) was gently added using a pipette. Vials were placed into black 50 ml Falcon tubes and mounted onto an overhead turning wheel (program C3, 15 rpm, Neolab, intelli-mixer). Solutions were exchanged every hour with 70%, 80% and 100% THF. Samples were incubated in 98% dibenzylether (DBE) for another 15 min (Sigma Aldrich, Steinheim, Germany) as last clearing solution. To avoid degradation of the fluorescent signal samples were imaged immediately after the clearing procedure. The cleared tumour and spleen were scanned with a commercially light sheet microscope (LSM) (LaVision BioTec GmbH, Bielefeld, Germany) by using a 2.0x magnification with a 2x objective lens and a white light laser with a wavelength spectrum ranging from 400 to 2400 nm. For the detection a filter with excitation range 470/24 and emission range 525/50 was applied.

We acquired z-stack measurements with 5 μm step size and a total range of 200 μm from the whole tumour material. In addition, we imaged single pictures from the tumour in a tumour depth of 175 μm and generated tagged image file format (tiff) or we converted the z-stack in an Audio Video Interleave (AVI) movie format. For AVIs of whole tumour z-stacks we used 40 pictures and 20 frames/second and a x-resolution of 720 and y-resolution of 576.

### Statistics

Statistical analysis was performed using GraphPad Prism v. 6.01 for Windows (GraphPad Software, La Jolla, CA, USA). Unpaired two-tailed t-Tests were used to determine the significant difference between the groups in *ex vivo* and *in vivo* comparison. Unless otherwise stated all values were calculated as mean ± standard deviation (SD).

## Additional Information

**How to cite this article**: Krüwel, T. *et al.* *In vivo* detection of small tumour lesions by multi-pinhole SPECT applying a ^99m^Tc-labelled nanobody targeting the Epidermal Growth Factor Receptor. *Sci. Rep.* **6**, 21834; doi: 10.1038/srep21834 (2016).

## Supplementary Material

Supplementary Figure S1

Supplementary Video S2

## Figures and Tables

**Table 1:** Overview of radiolabelling parameters.

	anti-EGFR	
	nanobody (^99m^Tc-D10)	IgG_1_ antibody (^99m^Tc-Cetuximab)
Labelling method	[^99m^Tc(CO)_3_]^+^ on 6 × His tag	modification with HyNic/tricine as coligand
Site-specific	Yes	No
Specific activity	183 ± 35 MBq/nmol	1700 ± 105 MBq/nmol
Radiochemical yield	48.8% ± 7.0	44.1% ± 10.1
Radiochemical purity	97.7% ± 1.2	97.3% ± 0.4

**Table 2:** *In vivo* and *ex vivo* determined tumour and tissue uptake of anti-EGFR nanobody ^99m^Tc-D10 and ^99m^Tc-Cetuximab.

Tumour model	Antibody	N	Tumour size mm^3^	Tumour uptake		Tissue uptake		Ratios		
				*ex vivo* % ID/g	*in vivo* % ID/cm^3^	*ex vivo* % ID/g	*in vivo* % ID/cm^3^	Tumour to blood *ex vivo*	Tumour to tissue *ex vivo*	Tumour to tissue *in vivo*
A431	^99m^Tc-Cetuximab	5	40.0 ± 38.9	6.5 ± 2.0	2.1 ± 0.8	0.43 ± 0.1	0.5 ± 0.2	0.9 ± 0.3	15.2 ± 4.1	8.4 ± 7.2
A431	^99m^Tc-D10	6	26.6 ± 16.7	2.3 ± 0.7	1.0 ± 0.6	0.14 ± 0.1	0.03 ± 0.01	12.1 ± 3.5	25.6 ± 18.8	36.2 ± 20.9
MDA-MB-468	^99m^Tc-D10	5	56.5 ± 21.2	1.3 ± 0.3	0.6 ± 0.2	0.15 ± 0.1	0.02 ± 0.02	5.4 ± 1.4	12.5 ± 7.3	42.8 ± 27.1
MDA-MB-231	^99m^Tc-D10	5	124.0 ± 102.3	0.25 ± 0.1	n.a.	0.06 ± 0.02	0.02 ± 0.01	1.1 ± 0.5	n.a.	n.a.

*In vivo* tumour and tissue uptakes were determined 45 min post ^99m^Tc-D10 and 24 h post ^99m^Tc-Cetuximab intravenous injection in tumour bearing mice by *in vivo* SPECT scans. *Ex vivo* tumour and tissue uptakes were determined after dissection following *in vivo* SPECT, approx. 100 min post ^99m^Tc-D10 and 25 h post ^99m^Tc-Cetuximab injection. Tumour sizes were determined by contrast enhanced CT scans. Muscle was used for calculating *ex vivo* tissue uptake. For *in vivo* SPECT scans, a region with the same size like the tumour containing tissue only was segmented on the contra lateral side and was used for determination of the *in vivo* tumour to tissue ratios. *In vivo* and *ex vivo* uptakes were expressed as percent of injected dose per cubic centimetre (% ID/cm^3^) and gram (% ID/g), respectively. Data are shown as mean ± standard deviation. n.a. = not applicable.
