# Supplementary material for: In vivo detection of small tumour lesions by multi-pinhole SPECT applying a 99mTc-labelled nanobody targeting the Epidermal Growth Factor Receptor
Source: Sci Rep. 2016 Feb 25;6:21834. doi: 10.1038/srep21834 (PMC4766429; doi:10.1038/srep21834)
Supplement: Supplementary Figure S1 [file srep21834-s1.pdf]

In vivo detection of small tumour lesions by multi-pinhole SPECT applying a ^{99m}Tc-labelled nanobody targeting the Epidermal Growth Factor Receptor

Thomas Krüwel [1], Damien Nevoltris [2], Julia Bode [3], Christian Dullin [1], Daniel Baty [2], Patrick Chames [2,#], and Frauke Alves [1,4,5,#,*]

[1] Department of Diagnostic and Interventional Radiology, University Medical Center Goettingen, Robert-Koch-Str. 40, 37075 Goettingen, Germany; thomas.kruewel@med.uni-goettingen.de (T.K.); christian.dullin@med.uni-goettingen.de (C.D.), falves@gwdg.de (F.A.)

[2] Antibody therapeutics and Immunotargeting, CRCM, Inserm U1068, Institut PaoliCalmettes, Aix-Marseille Université UM 105, CNRS UMR7258, F-13009, Marseille, France; d.nevoltris@gmail.com (D.N.); daniel.baty@inserm.fr (D.B.); patrick.chames@inserm.fr (P.C.);

[3] Molecular Mechanisms of Tumour Cell Invasion (V077), German Cancer Research Center, Im Neuenheimer Feld 581, 69120 Heidelberg, Germany; j.bode@dkfz.de (J.B.)

[4] Department of Haematology and Medical Oncology, University Medical Center Goettingen, Robert-Koch-Str. 40, 37075 Goettingen, Germany

[5] Molecular Biology of Neuronal Signals, Max-Planck-Institute for Experimental Medicine, Hermann-Rein-Str. 3, 37075 Goettingen, Germany

[#] These authors contributed equally as senior authors

* Author to whom correspondence should be addressed; E-Mail: falves@gwdg.de;
Tel.: +49 551 3899 0 (ext. 655); Fax: +49 551 3899 644

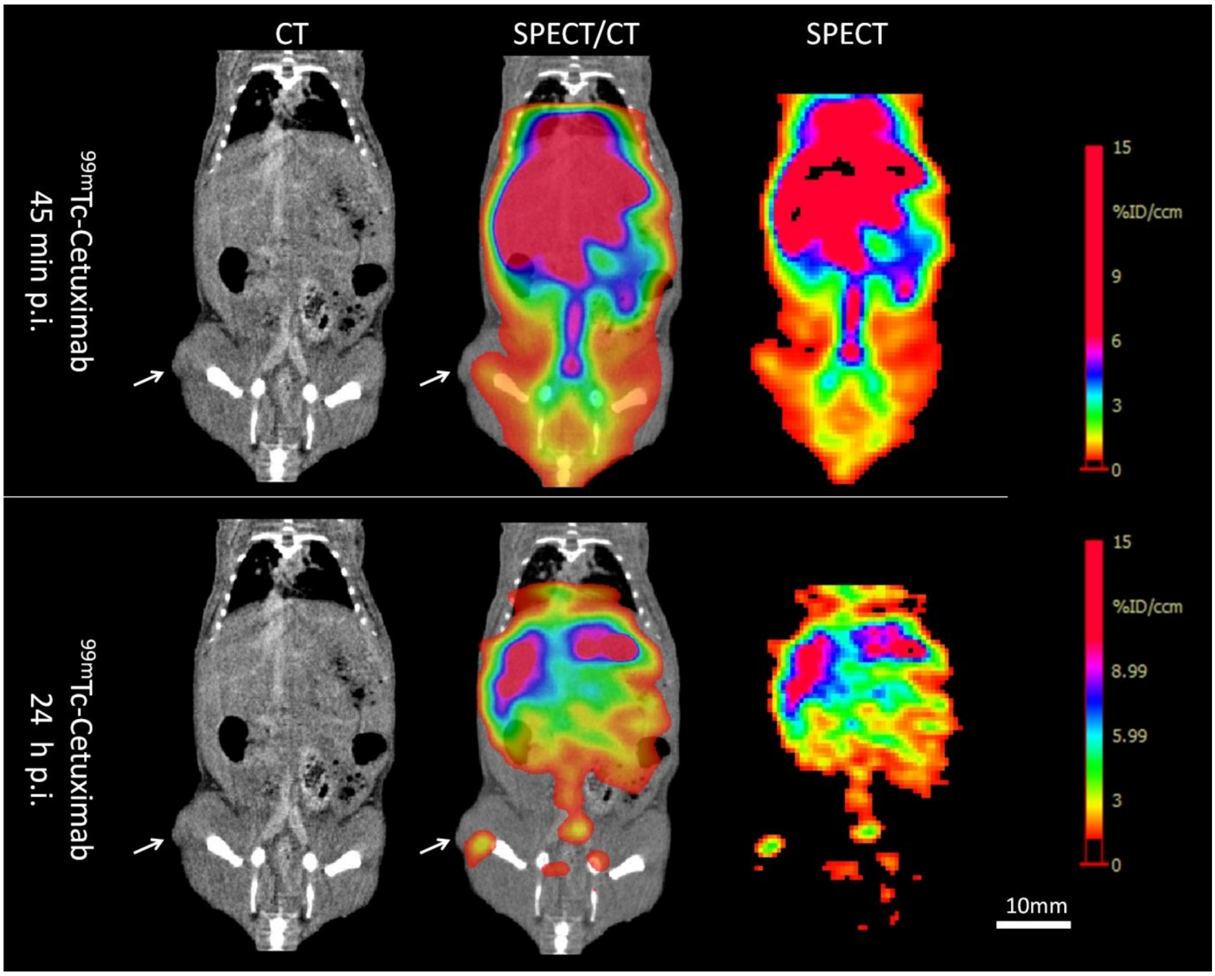

Supplementary Figure S1: *In vivo* tumour visualization of small human A431 tumour xenografts with anti-EGFR antibody ^{99m}Tc-Cetuximab by SPECT and in combination with CT at certain time points. Representative images of mice bearing a subcutaneous A431 tumour are shown, that received 9 pmol (10.5 MBq; tumour volume 16 mm^3) of the radiolabelled anti-EGFR antibody ^{99m}Tc-Cetuximab. SPECT imaging was performed 45 min and 24 h post intravenous probe injection. Contrast-enhanced CT and SPECT scans were performed on different modalities and images were aligned by hand according to ^{99m}Tc-pertechnetate landmarks (<30 kBq). Tumour is indicated by white arrows. No accumulation of ^{99m}Tc-Cetuximab in the tumour could be observed 45 min post injection due to high background signals. High uptakes were found in all organs and large blood vessels like the inferior vena cava and the external iliac veins. After 24 h the tumour could be visualized with ^{99m}Tc-Cetuximab, however still with a high background signal in the surrounding tissue.

Supplementary Video S2: Homogenous tumour penetration of the anti-EGFR nanobody D10 in an A431 tumour. Anti-EGFR nanobody AF488-D10 (14 pmol) was injected in the tail vein of a nude mouse bearing a A431 tumour. The movie depicts z-stack measurements with 5 µm step size and a total range of 200 µm from the whole tumour material at 4x magnification. The bright green spots in the middle of the tumour show the nanobody AF488-D10 binding on single cells.
